# Is 1:1000 adrenaline as a topical haemostat an effective alternative to control bleeding in dentistry and oral surgery?

**DOI:** 10.1038/s41415-023-6010-7

**Published:** 2023-07-14

**Authors:** Raj D. Aslam, Jonathan Liew, Eleni Besi

**Affiliations:** 41415338975001 Dental Core Trainee 2 in Oral Surgery, Edinburgh Dental Institute, UK; 41415338975002 Specialty Registrar in Oral Surgery, St Luke´s Hospital, UK; 41415338975003 Senior Clinical Lecturer and Honorary Consultant in Oral Surgery, Queen Mary University of London, Barts and The London School of Medicine and Dentistry, UK

## Abstract

Minor oral surgery can give rise to bleeding intra- and post-operatively. This can be minimal in most patients; however, it can be more problematic in patients with impaired clotting disorders, liver disease, or taking any form of anticoagulation therapy. Haemostatic agents are available to overcome such bleeding risks. The topical application of 1:1000 adrenaline used in medicine can be considered for use in dentistry. Adrenaline is widely used within medicine, surgery and dentistry. Examples include its use in local anaesthetic agents, in the management of anaphylaxis and as part of the cardiopulmonary resuscitation algorithm. 1:1000 adrenaline used topically for ear, nose and throat surgeries has shown improved visual fields during surgery with better surgical outcomes. It is relatively safe, but in patients with cardiovascular comorbidities, it should be practised with caution. This has precipitated the idea for its use within oral surgical procedures, including canine exposures, third molar surgery, root displacement into the antrum and periapical surgery. The haemostatic effect of 1:1000 adrenaline as an alternative should be considered in operative dentistry and oral surgery to aid in haemostasis and improve intra-operative visualisation, reducing the risk of iatrogenic damage and bleeding, thereby improving treatment outcomes.

## Key points

- This paper outlines the current haemostat agents used within medicine, surgery, and dentistry.
- 1:1000 adrenaline can be used as a topical haemostat which has been practised in otorhinolaryngology to control bleeding.
- This paper focuses on the prospects of using 1:1000 adrenaline topically in operative dentistry and oral surgical procedures.

## Introduction

Minor oral surgery can be associated with some post-operative complications, which could be based on a multitude of factors. Principally, one common post-operative complication in oral surgery is bleeding.^1^ Procedures including, but not limited to: raising mucoperiosteal flaps; dental extractions; dental implant placement; alveoloplasty; bone augmentation; and oral soft tissue surgeries, can give rise to intra- or post-operative bleeding. Bleeding from surgery can be linked to risks of morbidity and even mortality.^1^^,^^2^ With any minor oral surgical procedure, a patient's dental and medical background should be considered during the treatment planning phase. In most patients, post-operative bleeding is minimal and doesn't usually require further intervention. However, there are cohorts of patients with increased bleeding risks. This is often due to the patient's medical background, for instance: bleeding disorders, such as Von Willebrand disease or haemophilia; an underlying systemic condition, such as liver disease impairing clotting capabilities; or a recent surgery or co-morbidity that requires long-term anticoagulant or antiplatelet therapy.^3^ It is, therefore, prudent to have a sound knowledge of haemostatic measures available and to continuously keep abreast with evidence-based practice to manage such post-operative complications.^1^ The focus of this paper is to review the use of topical application of 1:1000 adrenaline as a haemostatic agent and whether it can act as a viable option within the field of dentistry/oral surgery.

Hiroshi *et al.*^4^ reported a 0.39% bleeding risk following dental extractions in patients not taking any form of anticoagulation. The same paper also showed an increased risk of post-operative bleeding in patients taking anticoagulants dabigatran, rivaroxaban, and warfarin, with percentage values of 1.65%, 3.41% and 3.63%, respectively. This extrapolates to a four-fold increased risk in patients on dabigatran, and a nine-fold increase in patients on rivaroxaban and warfarin, in comparison to patients not on any form of anticoagulation. Like rivaroxaban, apixaban and edoxaban are factor Xa inhibitors. The incidence rates of post-extraction bleeding in patients on direct oral anticoagulants (DOACs), vitamin K antagonists (VKAs), or patients not on anticoagulation, have been quoted to be 10.4%, 12% and 0.9%, respectively.^5^ A retrospective cohort study by Kataoka *et al.*^6^ assessed 258 patients who had dental extractions while on warfarin, with some patients also taking additional antiplatelet agents. The same study showed 8.1% of these patients were observed to have bleeding following dental extractions, which were controlled with haemostatic measures, such as pressure with gauze, infiltration of local anaesthetic with adrenaline, application of haemostatic agents, or suturing of the wound.^6^ These studies highlighted the possibility of post-operative bleeding following minor oral surgery in patients taking oral anticoagulation. Patient groups at increased risk of bleeding, especially those on VKAs or DOACs, may require additional haemostatic measures to reduce the risk of bleeding complications if local measures fail.^7^

With more patients being placed on DOACs, plus no quantitative tests to assess levels of anticoagulation, unlike warfarin, attendance to the emergency department because of post-operative bleeding can become more common.^8^^,^^9^ The Scottish Dental Clinical Effectiveness Programme stipulated the importance of categorising a patient's bleeding risk with recommended guidelines to manage patients on anticoagulant or antiplatelet therapy. This involves assessment of their medical status, treatment scheduled early in the day/week, use of haemostatic agents intra-operatively, interruption of their anticoagulant therapy based on the bleeding risk of the procedure and form of anticoagulant medication they receive.^8^

**Fig. 1:**
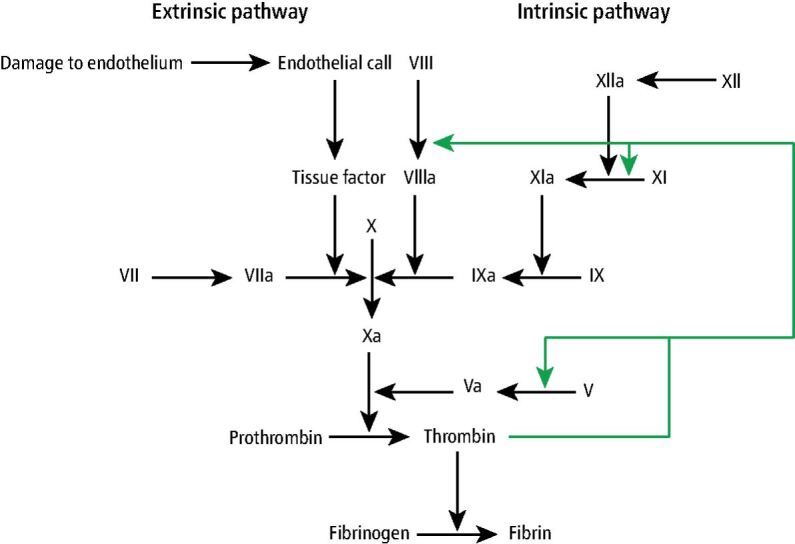
Clotting cascade^12^

The mechanism of haemostasis can be subdivided into primary and secondary. Primary haemostasis involves vasoconstriction of the damaged blood vessel and platelet plug formation. The damage to blood vessels triggers an endothelial constriction resulting in narrowing, thereby reducing blood flow. Damage to the endothelial layer during initial injury exposes the collagen sublayer, which initiates platelet adhesion, activation and aggregation, subsequently forming the platelet plug. The platelet plug creates a preliminary barrier to the injury.^10^ Secondary haemostasis involves the activation of a series of zymogens in an interlinked complex cascade. This ultimately leads to the conversion of fibrinogen to fibrin which forms part of the fibrin clot.^11^ A diagrammatic representation of the clotting cascade can be found in Figure 1.

Currently accepted approaches in the management of bleeding in the literature can be classified into local and systemic interventions.^13^ For most patients undergoing routine dental extractions with no background history of coagulopathy, simple pressure with the patient biting onto gauze post-operatively would achieve haemostasis.^14^ Suturing of the extraction socket to ligate vessels and tamponade bleeding can be an additional measure.^13^ Resorbable oxidised cellulose-based materials can also be used, as these materials contain a matrix that promotes platelet adhesion leading to fibrin clot formation. Gelatin sponges are an alternative to oxidised cellulose-based materials, where they are placed in sockets post extraction, acting as a scaffold for overlying blood clot formation.^14^^,^^15^ Haemostatic agents in different formulations, including adrenaline, ferric sulphate, silver nitrate and tranexamic acid, can also be applied to arrest uncontrolled bleeding. Bone wax can be applied to act as a barrier to areas of bleeding bone, so this becomes self-limiting. Cautery can be useful to aid haemostasis of soft tissues, whereby an electrical current is used to generate heat to seal and arrest bleeding blood vessels.^14^

Systemic interventions (Table 1) are generally facilitated in a secondary care setting. These include fresh frozen plasma, blood platelet concentrates, clotting factor replacement therapy, intranasal desmopressin, intravenous vasopressin, oral or intravenous tranexamic acid, and oral or intravenous epsilon amino-d-caproic acid.^13^^,^^16^ These interventions are often prescribed by colleagues in haematology.

**Table 1:** Local and systemic haemostatic agents^13^^,^^14^^,^^16^

Local	Systemic
Pressure with saline-soaked gauze	Fresh frozen plasma
Suturing the socket, ligation of vessels	Clotting factor replacement therapy
Resorbable oxidised cellulose products/Surgicel - matrix foundation promoting platelet adhesion and fibrin clot formation	Blood platelet concentrates
Gelatine sponges - promotes intrinsic clotting pathway	Intranasal desmopressin
Haemostatics agents - adrenaline, tranexamic acid, silver nitrate, ferric sulphate	Intravenous vasopressin
Bone wax	Oral or intravenous tranexamic acid
Cauterisation - electrical current to generate heat	Oral or intravenous epsilon amino-d-caproic acid

## The use of adrenaline in dentistry and medicine

Adrenaline is a vasoconstrictor that can be used topically to provide haemostasis. It is a catecholamine that acts on alpha- and beta-adrenergic receptors. Stimulation of the alpha-1 receptors can produce multiple effects on the sympathetic nervous system. Principally related to haemostasis, the activation of this receptor increases arteriole smooth muscle contraction, reducing the rate of blood flow.^17^^,^^18^

Adrenaline comes in different concentrations with differing degrees of effect. The standard local anaesthetic ampoule contains 2% lignocaine with 1:80,000 adrenaline and is the most commonly used preparation within dentistry. This indicates one part of adrenaline in every 80,000 parts of solution.^19^ Articaine is also a popular local anaesthetic choice with a formulation of 4% with 1:100,000 adrenaline.^20^ A systematic review by Nesbitt *et al.*^21^ postulated that using a more concentrated topical form of adrenaline could be beneficial intra-operatively to control bleeding.^22^^,^^23^ However, adrenaline would have to be administered with care, as systemic absorption in large quantities can lead to the presentation of undesirable cardiovascular complications, especially in those patients with cardiovascular comorbidities. Theoretical complications of using topical adrenaline may include arrhythmias, tachycardia, cerebrovascular accident, or even myocardial infarction, which is potentially fatal if not treated in time.^21^^,^^24^

Adrenaline is an important medication used within the medical emergency sector. Anaphylaxis is a life-threatening hypersensitivity response to trigger factors, such as venom (for example, insect bite), food (for example, peanuts), or certain medications/drugs (for example, penicillin). These triggers can result in the activation of inflammatory pathways, causing smooth muscle contraction and oedema in the airways, fluid extravasation leading to hypovolemia, and reduced cardiac function. If not handled promptly, anaphylaxis can have serious implications that can compromise a patient's airway, breathing, and/or circulation.^25^ The Resuscitation Council UK recommends the administration of 0.5 ml of adrenaline in 1:1000 concentration intramuscularly for the treatment of anaphylaxis in adults or children over 12 years.^25^ The General Dental Council mandates its registrants to adhere to the Resuscitation Council UK medical emergency guidance in dental practice, and an annual update of medical emergency and basic life support is compulsory as part of continued professional development.^26^^,^^27^ The Care Quality Commission also states that dental practices should stock the necessary emergency equipment and drugs as per Resuscitation Council UK guidance should a medical emergency arise; adrenaline 1:1000 concentration is one of the required drugs in the emergency kit.^28^

Adrenaline can also be used as part of cardiopulmonary resuscitation. The stimulation of alpha-1 adrenergic receptors on vascular smooth muscles initiates vasocontraction, thus increasing the aortic diastolic pressure, leading to the increase in coronary and cerebral perfusion pressure. This allows the return of spontaneous circulation.^29^ Adrenaline was historically documented in the literature for use in patients with acute asthma subcutaneously/intramuscularly.^30^ Beta-2 agonists are now the main treatment of choice for acute asthma; however, the Global Initiative for Asthma guidelines recommend intramuscular adrenaline for acute asthma in conjunction with angioedema or anaphylaxis only.^30^^,^^31^ Stimulation of the alpha-1 and beta-2 adrenergic receptors can reduce oedema and cause bronchodilation through bronchial smooth muscle relaxation, respectively.^30^

## The use of 1:1000 adrenaline in otorhinolaryngology

Clinicians within the otorhinolaryngology sector frequently use topical application of adrenaline 1:1000 in endoscopic sinonasal surgery.^32^ This typically involves the application of a cotton pledget soaked in 1:1000 adrenaline and applied directly to the sinonasal mucosa to aid in haemostasis.^32^ A literature review by Kuan *et al.*^22^ investigated the safety of 1:1000 adrenaline for haemostasis in endoscopic sinus surgery and concluded that topical application of adrenaline 1:1000 is relatively safe for use in endoscopic sinus surgery, with the recommendation to drain excess fluid out of the cotton pledgets before use. Kuan *et al.*^22^ also stressed that the cautious use of 1:1000 adrenaline is important in patients with cardiovascular comorbidities, including coronary artery disease, hypertension and arrhythmias.^22^

A cross-sectional study conducted by Peleman *et al.,*^33^ who investigated the haemodynamic changes following topical application of 1:1000 adrenaline before and during endoscopic sinus surgery, also concluded that the wrung-out adrenaline-soaked cotton pledgets used did not lead to concerning elevations, nor any electrocardiogram abnormalities, both pre- and intra-operatively. The same conclusion is further supported by Yim *et al.*^24^ All these studies confirmed that the topical application of adrenaline 1:1000 is a relatively safe method in otorhinolaryngology surgery to control bleeding and achieve haemostasis.

The advantages of 1:1000 topical application of adrenaline over its less concentrated forms can improve visualisation intra-operatively during surgical procedures. Dow *et al.*^34^ further demonstrated that a concentration of 1:1000 adrenaline created a more optimal visual field for the operator, which subsequently leads to better surgical outcomes, compared with the application of a 1:10,000 adrenaline concentration. This is further confirmed in another study by Korkmaz *et al.,*^35^ where the authors concluded that 1:1000 adrenaline is safe and effective in different endoscopic endonasal procedures, with its haemostatic properties enabling optimal operative times and minimal blood loss.

The literature collectively highlights and supports the safe and effective use of 1:1000 adrenaline as a topical method of application to aid haemostasis in sinonasal surgery. The application of 1:1000 adrenaline is scarce in dentistry, and to the authors' knowledge, there is limited literature that supports its use in operative dentistry. With the benefits shown from the use of 1:1000 adrenaline in ear, nose and throat surgery, the plausibility of similar applications of topical adrenaline 1:1000 to control bleeding in minor oral surgical or dental procedures, both intra- and post-operatively, is worth exploring.

## The use of 1:1000 adrenaline in oral surgery

Exposure of impacted canines can present with multiple intra-operative issues, such as difficulty in visualisation and moisture control during bonding secondary to bleeding. The aims of canine exposures can be three-fold: removal of hard and soft tissue obstructions; minimising damage to the surrounding tissue and avoiding exposure of the cementoenamel junction; and provide sufficient access to aid traction and alignment in orthodontics.^36^ However, the success of treatment outcomes can be limited by intra-operative bleeding where the surgeon's visualisation is obscured, complicating the operative procedure and delaying the operative time. Bleeding also creates a hindrance to a moist-free environment, reducing the success of bonding protocols.^37^ The topical application of 1:1000 adrenaline is effective in improving the operator's visual field and subsequently optimising the operating time by reducing intra-operative bleeding. This is not only pivotal in determining the success of bonding protocols, it is also critical in the provision of clear visual fields in the prevention of iatrogenic damage to surrounding structures and teeth.

Surgical extraction of third molars can potentiate intra- and post-operative bleeding, with the reported risk ranging from 0.2-5.8%.^15^ During surgical extractions, bone removal and sectioning of teeth with the use of a surgical drill can sometimes induce bleeding due to trauma to the medullary cavity of bone or pulp chamber of a tooth.^38^ This can result in impaired visualisation intra-operatively, reducing the operator's ability to assess the field sufficiently and to safely proceed with the surgical procedure. The use of 1:1000 adrenaline topically to the area of haemorrhage allows vasoconstriction and aids in the provision of a clearer operative field. Post-operatively, the application of 1:1000 adrenaline wrung-out gauze, as advocated in the otorhinolaryngology field, can be suitably applied if local measures have been insufficient.^34^ This can be a simple yet effective method of achieving post-operative haemostasis without the need for additional materials/products.

Root displacement into the maxillary antrum is a complication that may occur during the extraction of maxillary posterior teeth. Surgical approaches widely used include access through the created oroantral communication via the extraction socket, or the Caldwell-Luc technique with access through the anterior maxilla distal to the canine fossa via a vestibular approach.^39^ Both techniques have the potential to induce bleeding from soft tissues. Topical 1:1000 adrenaline can provide a clearer field of view through the arrest of haemorrhage in the confined sinus cavity, easing the surgeon's task of root identification and removal.

Bleeding from the soft tissue or bone during periapical surgery, or enucleation of a cyst, may impact the surgeon's visualisation, consequently affecting the surgical outcome and the quality of retrograde restoration. A randomised prospective study conducted by Menéndez-Nieto *et al.*^40^ compared the haemostatic effect of aluminium chloride versus 1 mg/ml (1:1000) adrenaline-soaked gauze in periapical surgery. Interestingly, aluminium chloride was found to produce adequate haemostasis in 72.5% of cases in this study compared to adrenaline, with a statistically significant difference of p <0.05. Even though these results indicate the superior haemostatic effect of aluminium chloride in periapical surgery, there are some limitations to its general use within dentistry. Aluminium chloride must be thoroughly irrigated from the surgical site, as failure to remove aluminium chloride after use can produce an inflammatory tissue reaction and therefore delay bone formation.^40^^,^^41^ Even though aluminium chloride can be rinsed off relatively easily with a saline flush, residue of product can remain in the bone.^41^ Hence, additional intervention is required to clean the surgical site following aluminium chloride application with a bone curette, and also the use of a surgical bur to clean the bony crypts.^40^^,^^41^ This makes the use of aluminium chloride more technique-sensitive and has the potential to induce local inflammatory responses, therefore compromising the healing of surrounding tissue if it is not thoroughly rinsed off.

Application of topical 1:1000 adrenaline during exposure of impacted canines, surgical extraction of third molars, retrieval of roots in the maxillary sinus, and periapical surgery is being carried out in the Oral Surgery Department at Edinburgh Dental Institute. The authors have found that it is very productive in arresting haemorrhage, providing a better field of vision in oral surgical procedures and thus optimising treatment success, efficiency and outcomes. A prospective multicentre study could help to achieve further quantifiable data on the use of 1:1000 adrenaline in oral surgery and operative dentistry.

## Other uses of adrenaline in general dentistry

Within the prosthodontic sector, an accurate impression of a prepared tooth for an indirect restoration is imperative to create a successful prosthesis. For subgingival crown margins, it is crucial to access these margins through the use of retraction cords, allowing the practitioner to obtain the tooth emergence profile.^42^ Retraction cords soaked in 1:1000 adrenaline can provide better control of gingival bleeding to aid in impression quality and improve the aesthetic outcome.

Adrenaline concentrations in 1:80,000 and 1:100,000 are readily available in local anaesthetic cartridges in dentistry and have been widely used by the dental profession daily. Operators often utilise adrenaline in local anaesthetic to arrest uncontrolled bleeding during an operative procedure. Due to the diluted formula of adrenaline, this can sometimes be counterproductive, with the potential to administer extra dose of local anaesthesia systemically. As Dow *et al.*^34^ alluded to, 1:1000 adrenaline applied locally in wrung-out gauze was found to be superior in its haemostatic capacity in contrast to a lower concentration of adrenaline, especially those found in local anaesthetic cartridges regularly used in dentistry.

The availability of 1:1000 adrenaline ampoules is a necessity since its primary use is for anaphylaxis treatment in a medical emergency.^28^^,^^43^^,^^44^ As previously discussed, for anaphylaxis, 0.5 ml adrenaline 1:1000 injection should be given intramuscularly.^25^ The use of adrenaline autoinjectors (0.15 ml or 0.3 ml 1:1000 adrenaline) is warranted if it is the only available alternative in an emergency.^43^^,^^44^

## The licensed use of adrenaline

The British National Formulary states the licenced use of adrenaline is limited to the following: cardiopulmonary resuscitation; treatment for anaphylaxis; control of bradycardia following myocardial infarction in arrhythmia patients that are unstable or at risk of asystole; and acute hypotension in neonates. This may vary among children.^45^ The topical use of adrenaline is not the intended primary use of this medicine. Nonetheless, the supportive evidence available in the literature has perpetuated its continuous successful use in varying parameters of application in dentistry and surgery.

## Cost of various market-available haemostatic agents

**Fig. 2:**
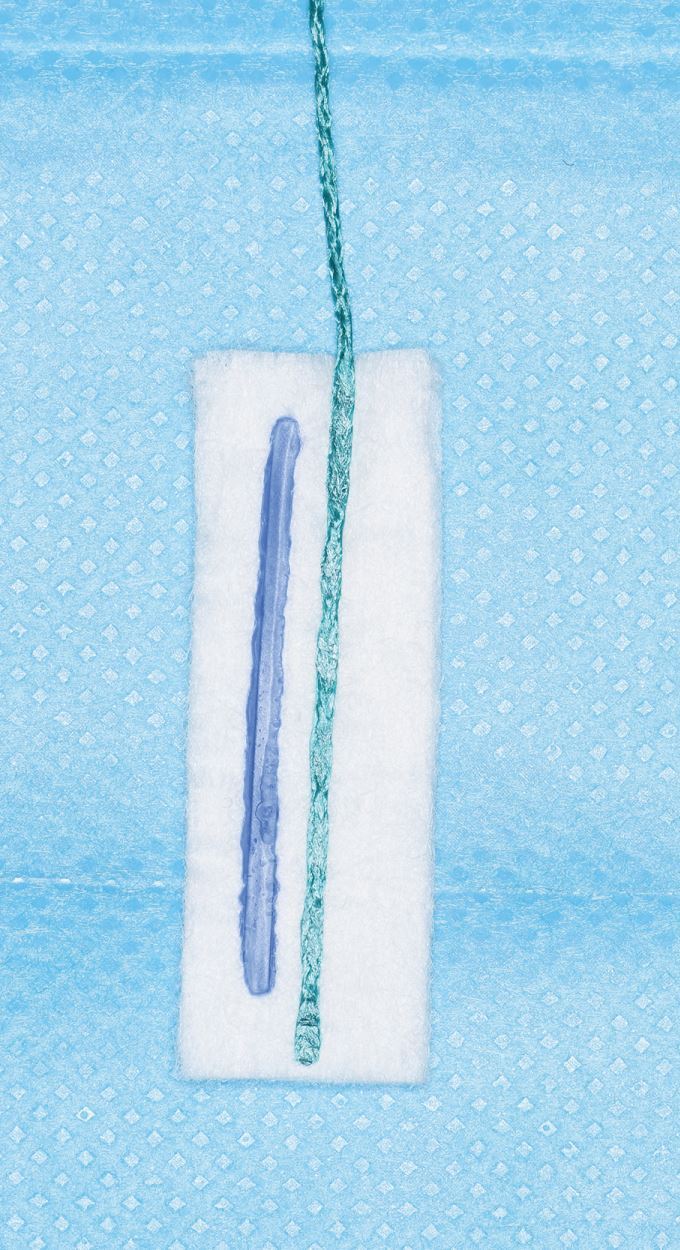
Surgical pattie

**Fig. 3:**
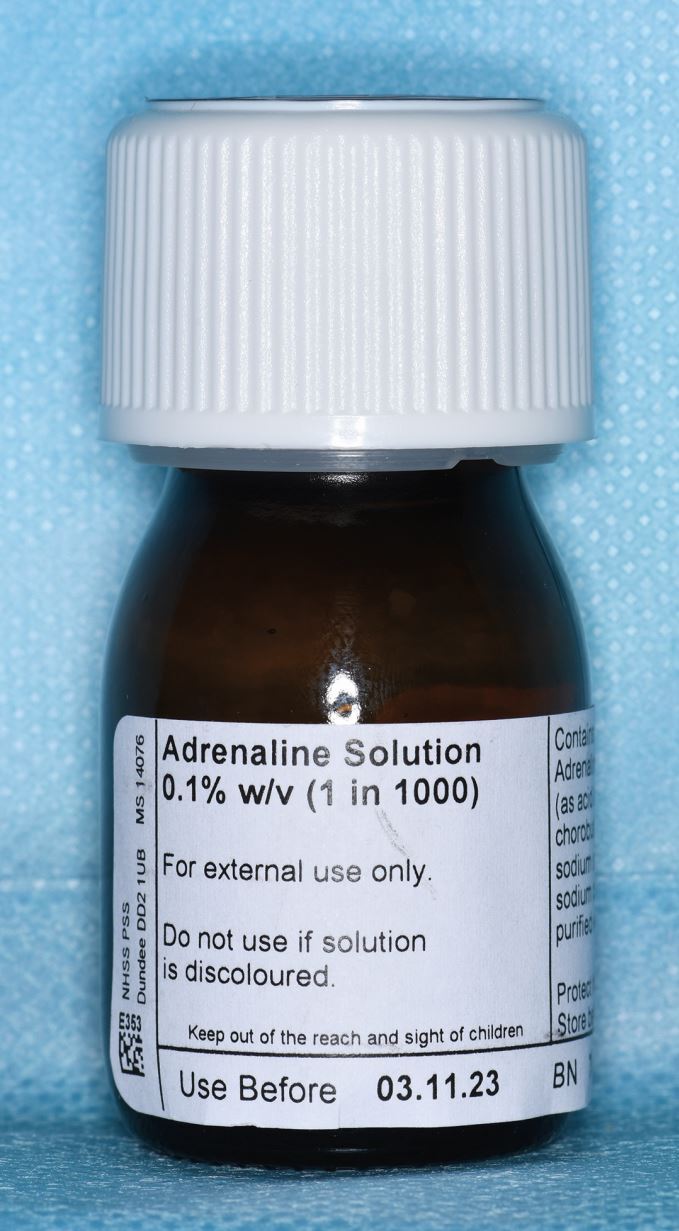
30 ml 1:1000 adrenaline

The cost of oxidised regenerated cellulose, Surgicel (Ethicon, Somerville, NJ) is approximately £9.40 for 1.35 x 5 cm per sachet and £11.41 for 5 x 7.5 cm per sachet. Surgical patties (Fig. 2) are cotton-based products primarily used in neurosurgery or otorhinolaryngology. They aid in haemostasis through the absorption of fluids or blood.^46^ They are available, with a box of 20 equating to approximately £109.53. The price of local dental anaesthetics routinely varies based on the suppliers. This could range from £25-30 for a box of 50 cartridges of 2% lignocaine with 1:80,000 adrenaline or 4% articaine with 1:100,000 adrenaline. 1:1000 adrenaline is available in ampoule, vial, or bottle form. This can be easily obtained from major suppliers in the market. For example, a 30 ml 1:1000 adrenaline solution (Fig. 3) can cost around £15.40 per bottle, whereas a pack of ten 1:1000 (1 mg/ml) adrenaline ampoules can cost approximately between £6-11 based on the supplier.

The price of 1:1000 adrenaline can be argued to be relatively expensive; however, its beneficial capacity and improved haemostatic effect can outweigh this, especially if improving clinical outcomes intra-operatively as supported in the literature. Therefore, where haemorrhage can be an issue (eg obscuring the visual field), its use can prove to be beneficial for the operator and so improve the clinical outcomes for the patient. The shelf life of this is approximately 18 months, therefore it can be stored for relatively long periods without the need for high turnover.

## Conclusion

The use of 1:1000 adrenaline topically in dental/oral surgical procedures intra- and post-operatively may provide an alternative solution in the arrest of haemorrhage. It has been shown to improve operative feasibility and increase the success of treatment outcomes in otorhinolaryngology. It should, however, be used judiciously in patients with cardiovascular comorbidities. It is, therefore, worth considering the use of 1:1000 adrenaline topically as an alternative to arrest bleeding complications, adding to the armamentarium of haemorrhage treatment modalities in oral surgery/dentistry. Further research with high-quality evidence is warranted to establish its benefits and effectiveness in medicine, surgery, and dentistry.
